# Building family caregiver skills using a simulation-based intervention for care of patients with cancer: protocol for a randomized controlled trial

**DOI:** 10.1186/s12912-021-00612-4

**Published:** 2021-06-09

**Authors:** Susan R. Mazanec, Eric Blackstone, Barbara J. Daly

**Affiliations:** 1 Frances Payne Bolton School of Nursing, Case Western Reserve University, Cleveland, Ohio USA; 2 Seidman Cancer Center, University Hospitals Cleveland Medical Center, Cleveland, Ohio USA

**Keywords:** Family caregivers, Caregivers, Simulation, Experiential learning, Cancer, Radiation therapy

## Abstract

**Background:**

Family caregivers of patients with cancer undergoing radiation therapy experience significant distress and challenges related to high symptom burden and complex care demands. This is particularly true for caregivers of patients with head and neck, esophageal, anal, rectal, and lung cancers, who are often receiving combined-modality treatment and may have tracheostomy tubes, gastrostomy tubes, or colostomies/ileostomies. This study aims to evaluate a simulation-based nursing intervention to provide information, support, and training to caregivers during radiation therapy.

**Methods:**

This randomized controlled trial will include a sample of 180 patients and their family caregivers. Caregivers assigned to the control group will receive usual care and an informational booklet from the National Cancer Institute (NCI). Those in the intervention group will receive usual care, the NCI booklet, and three meetings with a nurse interventionist during radiation treatment followed by a booster call two weeks posttreatment. Intervention sessions focus on themes consistent with the trajectory of radiation therapy: the patient experience/needs, the caregiver experience and dyad communication, and transition to survivorship. Outcomes are measured at baseline, end of treatment (T2), and 4 (T3) and 20 (T4) weeks posttreatment, with the primary outcome being caregiver anxiety at T4.

**Discussion:**

This trial is innovative in its use of simulation in a psychoeducational intervention for family caregivers. The intervention is administered at point-of-care and aimed at feasibility for integration into clinical practice. Patient quality of life and healthcare utilization measures will assess how providing support and training to the caregiver may impact patient outcomes.

**Trial registration:**

The trial was registered on 08/14/2019 at ClinicalTrials.gov (identifier NCT04055948).

## Background

Rigorous, intense, combined-modality cancer treatment (radiation, surgery, and/or chemotherapy) is associated with high symptom burden. Approximately 69 % of patients will report multiple symptoms with moderate-to-severe distress during treatment [1] and will experience persistent treatment side effects, debilitating functional impairment, and complex psychosocial issues. They will need to rely on family not just for emotional support, but for actual physical care tasks. Family caregivers, also known as informal caregivers, are essential to achieving positive patient outcomes and avoidance of complications, such as dehydration, malnutrition, and excessive symptom burden, yet few caregiver interventions have been implemented in clinical settings [2]. There is great need for caregiver interventions that are feasible and delivered at the point of care, promoting integration of the caregiver into the clinical setting, facilitating caregiver assessment, support, and training [3].

In this report, we share the protocol to test a psychoeducational intervention for family caregivers of patients with cancers of the head and neck (HNC), esophagus, rectum, anus, and lung (non-small cell lung cancer [NSCLC]) who are receiving radiation therapy with or without chemotherapy. These patients typically experience a high symptom burden associated with significant functional impairment. Recovery from treatment is prolonged, with persistent treatment and disease effects lasting years, well into survivorship for some patients [4–8]. Demands on family caregivers are substantial and often include quickly gaining new knowledge and technical skills to manage treatment side effects, medications, nutritional supplements, ostomies, and tracheostomy and gastrostomy tubes. Yet, in two studies of caregivers of patients with mixed cancer diagnoses, up to 58 % of caregivers reported unmet training needs [9, 10]. Beyond the hands-on care, caregivers must communicate with the patient, family, and healthcare providers about the illness. Communication skill building is essential to reduce “communication burden” in cancer family caregivers, described as the caregiver’s perceived communication challenges, including initiating discussions, sharing emotions and feelings, and providing information to others about the patient’s cancer [11]. There is high potential for communication burden in caregivers as they must adapt to an often dramatically altered lifestyle and changing roles within the family [12–15].

Although the literature describing the needs and experience of caregivers of individuals with HNC, lung, esophageal, rectal, and anal cancer is limited, it is clear that the early phase of the cancer trajectory, within the first 6 to 12 months following diagnosis, entails a significant time of stress for caregivers [16, 17]. Later-stage disease in particular and higher symptom burden, common with these cancers, are associated with increased caregiver psychological distress [18]. Studies specific to HNC caregivers underscore the severity of psychological problems: caregivers have significantly higher levels of anxiety than the patients during treatment [19], 40 % can be classified as having a clinical anxiety disorder [17], and approximately 15 % have a depressive disorder [20]. It is essential to intervene with caregivers early in the care trajectory, particularly during the treatment phase, to offer psychological support [21] and to prepare them for dealing with acute toxicities of treatment and the unique difficulties associated with these cancers. Caregivers have multiple unmet supportive care needs, most often related to fears of the patient’s decline, concerns about recurrence, and feelings about death and dying [22].

The purpose of this study is to measure the effect of a caregiver intervention that incorporates simulation techniques focused on skill development and communication to improve caregiver outcomes, increase self-efficacy for caregiving, and improve patient outcomes. Simulation, commonly used in training healthcare professionals, is a form of experiential learning that creates events or situations to mimic clinical situations [23]. The learner is able to solve problems, make decisions, and practice skills in a safe, supportive learning environment. Rarely used with cancer family caregivers, simulation has been shown to be effective in training parents of chronically ill children to manage seizures [24], home ventilators [25], and diabetes [26]. Communication skills training for family caregivers, most often noted in the dementia care literature, includes both didactic and role-play teaching methods and has been shown to be effective in improving caregiver communication skills, competencies, and knowledge [27].

Few studies were found that tested a simulation or experiential intervention with cancer caregivers. In a study of simulation use with caregivers of individuals with head and neck cancer, a one-hour, group-format tracheostomy education class that used an anatomical trainer was effective in reducing anxiety in family caregivers [28]. In another study, cancer caregiver training with an experiential learning component delivered in a single session in the inpatient setting was effective in producing short-term improvement in self-efficacy for managing patient symptoms and caregiver stress [29]. Our study differs from these studies in that the intervention is delivered in individual, private sessions with the caregiver in the practice setting, is tailored to the specific needs associated with each cancer type, is offered over the weeks of active treatment, allowing for real-time (point-of-care) attention to caregiver learning needs, and incorporates simulations for both technical and communication skills training with psychoeducational strategies.

The theoretical framework for this study is the revised Self- and Family Management Framework [30], which acknowledges the integral role of family in managing a chronic condition. The framework identifies three broad self-management processes of “focusing on illness needs,” “activating resources,” and “living with the condition.” These processes are influenced by a complex array of facilitators and barriers at the individual, family, community, and healthcare system levels. The processes lead to proximal outcomes such as behaviors, cognitions, biomarkers, and symptom management. These proximal outcomes are mediators of more distal outcomes of self-management (health status, quality of life, psychosocial status, and health care). Our intervention specifically focuses on one component of this framework by targeting the processes for family caregivers to increase self-efficacy, a cognitive proximal outcome, and to impact more distal caregiver and patient outcomes.

The intervention is based on self-efficacy, a key concept of Social Cognitive Theory. Perceived self-efficacy is the belief that one can successfully perform a specific behavior to produce an expected outcome, and according to Bandura, stronger self-efficacy beliefs can result in greater coping efforts to overcome challenging or threatening activities [31]. Caregiver self-efficacy for the complex tasks of caregiving has implications for both the patient and caregiver. In a study of 152 dyads of patients with lung cancer and their family caregivers, lower levels of caregiver self-efficacy for pain and symptom management was significantly associated with higher levels of caregiver strain and mood disturbance, as well as higher levels of patient-reported pain, fatigue, anxiety, and depression, and lower levels of patient-reported quality of life [32]. In the current study, strategies to strengthen self-efficacy for caregiving and self-care will occur through vicarious experience (observing nurse modeling behavior during simulation), performance accomplishments (repeated simulation practice sessions), verbal persuasion (supportive nurse communication during intervention), and attention to the caregiver’s emotional state (screening and intervention for emotional distress).

## Methods/design

### Aim

The specific aims are:

1. Evaluate the effect of the intervention, as compared to a control group, on caregiver anxiety, depression, health-related quality of life (HRQOL), and fatigue.
2. Measure the effect of the intervention, as compared to a control group, on patient outcomes (HRQOL and interrupted treatment course), and healthcare utilization outcomes (hospital admissions, emergency room visits, and use of intravenous [IV] fluids).
3. Determine if caregiver self-efficacy mediates the effect of the intervention on caregiver anxiety.
4. Determine if patient illness factors, care demands (hours per week spent caregiving), and patient and caregiver demographic factors moderate the relationship between the intervention and caregiver outcomes.
5. Compare the costs of healthcare utilization (unplanned hospital admission, unplanned emergency room visits, and unplanned use of IV fluids) between the intervention and control groups.

### Design & setting

**Fig. 1:**
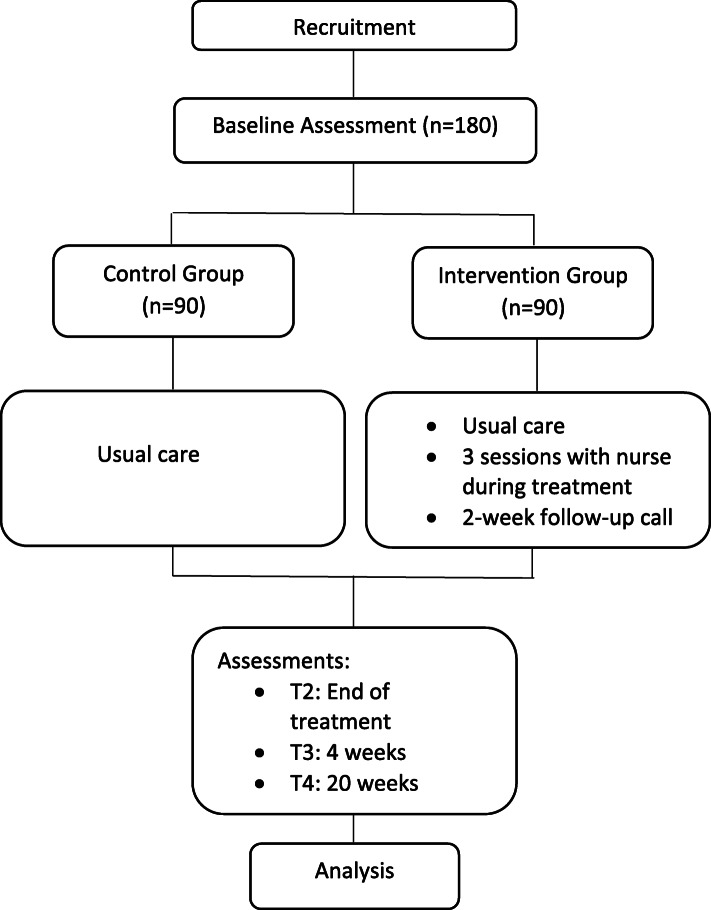
Flow chart for the Randomized Controlled Trial

The study is a randomized controlled trial with two groups. The intervention is delivered during the course of radiation therapy and involves three sessions with the caregiver, followed by a telephone booster contact two weeks after treatment. Patient and caregiver outcomes are measured at baseline, at the end of radiation treatment, and 4 and 20 weeks post-radiation treatment. The study is being conducted at the University Hospitals Seidman Cancer Center in Cleveland, Ohio, United States. The study was approved by the Institutional Review Board of University Hospitals Cleveland Medical Center (Study 20190943) and is registered at ClinicalTrials.gov NCT04055948. The flow chart for the randomized controlled trial is shown in Fig. 1.

### Participants

Using a convenience sampling methodology, radiation oncology clinic schedules are screened to identify eligible patients. Inclusion criteria for patients are: (1) 18 years of age or older. (2) Diagnosis of stage I, II, III cancers of the rectum and anus, stage I – IVA esophageal cancer; stage III NSCLC; and stage I – IV A/B HNC (tongue, gum, oral cavity, nasopharynx, oropharynx, hypopharynx, parotid, or larynx). (3) Receiving their first course of radiation therapy. (4) Has an identified family caregiver who is willing to participate. Inclusion criteria for caregivers are: (1) 18 years of age or older; (2) family member or friend of an adult patient described above; and (3) identified by the patient as his/her primary caregiver, who is providing daily assistance and/or emotional support. Exclusion criteria include patients without a caregiver or who are receiving hospice care. Caregivers who are themselves undergoing active cancer treatment are excluded (hormonal treatment allowed).

The study is presented to the patient prior to beginning radiation treatment, typically at the scheduled treatment planning visit in the clinic. The patient is asked to identify an adult family member or friend who is their primary caregiver. Once the patient has given approval to contact the caregiver, the research assistant contacts the caregiver to explain the study and invite participation. Once written or electronic consent is obtained, baseline measures are collected from the patient and caregiver. All caregivers are screened for health literacy using a 4-item Brief Health Literacy Screening Tool, a self-report tool that assesses comprehension to written and verbal health information [33]. The research team employs communication and teaching strategies from the Agency for Healthcare Research and Quality’s Health Literacy Universal Precautions Toolkit for individuals identified as having limited health literacy [34].

### Randomization & blinding

The project manager randomly assigns participants to one of two groups (control or intervention) in a one-to-one allocation ratio using a computerized minimization stratified randomization technique. Stratification variables are caregiver gender, caregiver age, and patient cancer type. Participants are informed of their group assignment by the project manager after baseline measures are collected. Caregivers in both groups receive usual care by non-study clinicians, which includes a weekly visit with the radiation oncologist and clinic nurse for the patient and caregiver. The weekly visit is brief (10–15 min) and focused on how the patient is tolerating the radiation treatments and management of any treatment side effects. Caregivers randomized to the control group receive the National Cancer Institute (NCI) booklet, *When Someone You Love is Being Treated for Cancer.* Caregivers randomized to the intervention group receive the same NCI booklet and also meet with the intervention nurse during radiation weeks 1 and 3, and at the end of radiation treatment (weeks 6–8). A telephone booster call is made 2 weeks after radiation therapy is completed. The research assistants, who collect data, are blinded to group assignment.

### Description of the intervention

The intervention sessions were designed to coincide with the typical patient experience during treatment and each has a corresponding theme. The themes of the intervention  sessions are: (1) the patient’s experience/needs, (2) the caregiver experience and the patient-caregiver relationship, and (3) the transition to post-treatment survivorship. Each session is standardized in that it begins with addressing any issues or concerns that the caregiver may be experiencing, assessing the caregiver’s level of distress, and providing emotional support. This is followed by provision of relevant information related to the theme of the session, followed by the simulation experience. The technical simulations with a tracheostomy tube, gastrostomy tube, colostomy/ileostomy, and skin/wound care use a low-fidelity manikin. The communication skills training uses common caregiving scenarios to stimulate discussion, identify barriers, and solve problems. Communication skills include asking questions, active listening, staying calm, expressing feelings, recognizing depressive feelings, and identifying resources for both the patient and caregiver. The self-care simulations include specific skills for caregiver self-care, including identifying strategies to reduce fatigue and improve sleep, prioritizing activities, taking time for leisure activities, finding support, and managing one’s own health care.

The simulation protocols were modeled after procedures used in nurse education. They have a standardized format: (1) caregiver learning objectives; (2) pre-simulation preparation, during which the intervention nurse shows the caregiver the manikin and reviews steps of the procedure; (3) basic simulation scenarios followed by more complex alternative scenarios that have a series of critical events that the caregiver responds to; and (4) a post-simulation debriefing during which the intervention nurse answers questions, assesses the caregiver’s confidence in performing the skills, and provides additional training as necessary. Each simulation manual includes a structured checklist with scenarios, expected caregiver behaviors, and nurse prompts. Although the simulation training follows a consistent outline, the content is tailored to the caregiver’s needs. Caregivers have the opportunity to repeat simulations if needed.

Nurse interventionists document any tailoring of the intervention to meet caregiver needs, caregiver’s engagement in the session, and caregiver verbalization of understanding of session content. The dose of the intervention is being tracked by recording the duration of each session, any additional contacts with the caregiver, as well as any missed visits by the caregiver.

### Study variables and measures

For patients and caregivers in both the intervention and control groups, study measures are obtained in-person, via mail, through email, or by telephone by the research assistant at baseline (T1), at the end of treatment (T2), and at 4 (T3) and 20 (T4) weeks post treatment (Table 1). The primary endpoint is caregiver anxiety at T4.

**Table 1:** Study Measures

Variable	Instruments	Time of measure			
		**T1**	**T2**	**T3**	**T4**
**Caregiver Outcomes**					
CG Anxiety	PROMIS Anxiety Short Form 7a	X	X	X	X
CG Depression	PROMIS Depression Short Form 8b	X	X	X	X
CG HRQOL	PROMIS Global Health Scale v. 1.0/1.1	X	X	X	X
CG Fatigue	PROMIS Fatigue Short Form 7a	X	X	X	X
CG Burden	Caregiver Reaction Assessment	X		X	
**Patient Outcomes**					
PT HRQOL	FACT-C, FACT-E, FACT-HN, FACT-L (v. 4)	X	X	X	X
Interrupted radiation treatment course (total number of missed days from treatments)	Radiation Therapy Record		X		
**Healthcare Utilization Outcomes**					
Hospital admissions	Hospital record		X	X	X
Emergency room visits	Hospital record		X	X	X
Intravenous fluid use	Hospital record		X	X	X
**Cognitive Mediator**					
CG self-efficacy for caregiving	Caregiver Inventory	X	X	X	X
	Investigator-constructed self-efficacy scale for managing side-effects and specific skills	X	X	X	X
**Potential Moderators (Covariates)**					
Patient Illness Factors (stage, time since diagnosis, treatment type)	Patient Medical Record	X			
Use of palliative care services	Patient Medical Record	X	X	X	X
Patient Performance Status	ECOG Performance Status	X	X	X	X
Care Demands (hours/week spent caregiving)	Survey questions (CG)	X	X	X	X
Demographic characteristics (race, marital status, employment, socioeconomic status)	Survey questions (CG and PT)	X			
Use of mental health services (support groups, social work, and/or psychologist)	Survey questions (CG and PT)	X	X	X	X

Note: *CG *Caregiver, *PT *Patient

#### Caregiver outcome variables

Anxiety is measured with the Patient-Reported Outcomes Measurement Information System (PROMIS®) Anxiety Short Form 7a [35]. This 7-item questionnaire assesses self-reported fear, worry, anxiety, tension, nervousness, and restlessness in the family caregiver over the last 7 days. Depression is measured with the PROMIS® Depression Short Form 8b [35]. This 8-item questionnaire assesses caregiver self-reported negative mood (sadness, guilt), views of self (worthlessness), and social cognition (loneliness), as well as decreased positive affect and engagement. It assesses depression over the last 7 days. HRQOL is measured using the PROMIS® Global Health Scale v.1.0/1.1, a 10-item questionnaire that evaluates global physical and mental health [35]. Caregiver fatigue is measured with the PROMIS® Fatigue Short Form 7a, a 7-item questionnaire that evaluates the self-reported experience of fatigue (frequency, duration, intensity) and the impact of fatigue on daily activities [35]. It assesses fatigue over the last 7 days. Caregiver burden is measured using the Caregiver Reaction Assessment (CRA), which assesses the subjective negative and positive impact of caregiving [36, 37]. The 24-item scale consists of five subscales: disrupted schedule, financial problems, lack of family support, health problems, and impact of caregiving on self-esteem.

#### Patient outcome variables

HRQOL in patients is measured using the disease-specific versions of the Functional Assessment of Cancer Therapy – General (FACT-G) [38] that include common HRQOL subscales (physical, social, emotional, and functional well-being) plus cancer-specific questions. The effect of the intervention on HRQOL across diagnoses will be analyzed using the common FACT subscales and will describe the symptom experience within each diagnostic group using the cancer-specific FACT subscale. The Functional Assessment of Cancer Therapy - Colorectal (FACT-C, Version 4) is a 37-item questionnaire that measures self-reported HRQOL in patients with colorectal cancer over the last 7 days [39]. The Functional Assessment of Cancer Therapy - Head and Neck Scale (FACT-H&N, Version 4) is a 39-item questionnaire that assesses self-reported HRQOL in patients with head and neck cancer over the last 7 days [40]. The Functional Assessment of Cancer Therapy - Esophageal (FACT-E, Version 4) is a 44-item questionnaire that measures self-reported HRQOL in patients with esophageal cancer over the last 7 days [41]. The Functional Assessment of Cancer Therapy - Lung (FACT-L, Version 4) is a 33-item questionnaire that measures self-reported HRQOL in patients with lung cancer over the last 7 days [42, 43]. Interrupted radiation treatment course, defined as the total number of missed treatment days due to patient or caregiver reasons, is determined from the patient’s radiation therapy treatment record.

#### Healthcare utilization outcomes

Healthcare utilization outcomes include hospital admission, emergency room visits, and IV fluid use for treatment of dehydration. We will evaluate the effects of the intervention, as compared to the control group, on these outcomes, as well as describe the costs associated with these outcomes. These outcomes will be measures at T2, T3, and T4.

#### Mediating variable

Caregiver self-efficacy for caregiving is measured with the Caregiver Inventory, a 21-item questionnaire that evaluates the domains of managing medical information, caring for the care recipient, caring for oneself, and managing difficult interactions and emotions [44]. This scale will be administered at all time points.

#### Covariates

Patient illness factors – stage of cancer, time since diagnosis, treatment type (chemoradiotherapy vs. radiotherapy alone), human papillomavirus status (in HNC and anal cancer), use of palliative care, and patient performance status – will be recorded on the enrollment form. Updates for use of palliative care and patient performance status will be noted at T2, T3, and T4. Care demands, defined as hours/week spent caregiving, will be assessed at all time points. Demographic characteristics of both the patient and caregiver will be collected at baseline (T1). Use of mental health interventions (support group participation, social work and/or psychologist involvement) will be assessed at all time points.

### Data management & confidentiality

Data is collected primarily online using Research Electronic Data Capture (REDCap; https://project-redcap.org), although paper data forms are also used when necessary. REDCap is an institution-specific secure web platform for managing data and surveys. The de-identified data from REDCap will be downloaded to a password-protected statistical database when ready for data analysis. All participant data entered into the statistical database will use an assigned code number. No names or identifying variables are stored with the actual data.

### Data analysis

An intent-to-treat analysis is planned. For Aim 1, a linear mixed model repeated measures analysis will be used to obtain mean changes from baseline in anxiety scores at Times T2, T3, and T4, using baseline anxiety as a covariate, also adjusting for covariates. Other caregiver outcomes will be compared between groups using a similar approach.

Aim 2 will measure the effect of the intervention, as compared to a control group, on patient outcomes (HRQOL, interrupted treatment course) and healthcare utilization outcomes (hospital admissions, emergency room visits, and IV fluid use). Most outcomes will be count variables enumerating the cumulative numbers at T2, T3, and T4, which can be analyzed using Poisson regression methods implemented as generalized linear models. HRQOL, a continuous measure, will be analyzed using a linear mixed model with repeated measures at T1, T2, T3, and T4. We will analyze the effect of the intervention on HRQOL across diagnoses using the common FACT subscales and will describe the symptom experience within each diagnostic group using the cancer-specific FACT subscale.

Aim 3 focuses on estimating and testing the mediating effects of family caregiver self-efficacy on their anxiety at Time T4 and other time points. The analysis will follow a standard approach of first testing the associations between intervention and the mediator (self-efficacy), and between self-efficacy and the outcome (anxiety) controlling for the intervention. If both associations are significant, the indirect effect of the mediator is estimated as the difference in the intervention effect on the anxiety before and after adjusting for the mediator [45–47].

Aim 4 examines if patient illness factors, care demands, demographic factors, and/or use of mental health interventions during treatment moderate the relationship between the intervention and caregiver outcomes. Effects of potential modifiers of the treatment effect will be examined by including terms for the modifier by treatment interaction effect in the basic ANCOVA model used to test the primary hypothesis.

For Aim 5, Medicare reimbursement rates will be used to estimate healthcare utilization costs in both intervention and control groups regardless of the age or insurance of the participant. Using the Diagnosis-Related Group (DRG), diagnosis and procedure codes we will determine what Medicare “would have paid” for those services, and will use that as a proxy for cost. Cost of IV fluids will be estimated using wholesale acquisition cost. We will compute a healthcare utilization cost for each participant. Mean differences between the two groups will be analyzed using a two sample *t*-test for independent means.

### Sample size

The power calculation for this study was based on Aim 1. With 180 subjects randomized and an estimated 10 % dropout rate, the sample size for the primary analysis is 162 caregivers (81 per arm). Assuming a correlation of 0.50 between caregiver anxiety scores at baseline and T4, when adjusting for baseline anxiety, a difference in means of 0.36 standard deviations (*SDs*) can be detected with 80 % power using a 2-sided test with significance level 0.05, where *SD* is the within-group standard deviation of anxiety scores at Time T4. This 0.36 effect size (Cohen’s d) is intermediate between a “small” (0.2) and “moderate” effect size, as defined by Cohen [48], and is close to the pooled estimate of 0.29 from a meta-analysis of interventions (psychoeducational, skills training, and therapeutic counseling) with family caregivers of cancer patients [2].

### Data and safety monitoring

 A Data and Safety Monitoring Committee has been formed for this study and consists of members outside the study team, including two faculty members from the Frances Payne Bolton School of Nursing, Case Western Reserve University and a physician from the Case Comprehensive Cancer Center. Study team members also include the principal investigator, project manager, and statistician. Twice annually, this committee will review data on this study regarding: (1) study safety including auditing selected cases for compliance with IRB requirements, (2) conformance with informed consent requirements, verification of source documents, and investigator compliance, (3) minimizing research-associated risk, and (4) protecting the confidentiality of participant data. The rate of recruitment refusal and subject attrition will be tracked and reported at these reviews. Differential attrition from all study groups will be monitored. If there are recommendations made by the Data Safety Monitoring Committee, the action plan for response or notice of any actions taken by the IRB regarding the research and any responses to those actions will be provided to National Cancer Institute officials within 2 weeks.

Although this study is deemed as having minimal risk, we recognize that some unanticipated or adverse events could occur. As they occur, all unanticipated events and adverse events are immediately reported to the principal investigator who will report them to the IRB according to the IRB protocol for both serious and non-serious adverse event and unanticipated problem reporting. These are summarized in reports to the Data Safety Monitoring Committee twice a year. Annual progress reports to the IRB and National Cancer Institute include a summary of the Data Safety Monitoring Committee’s activities and findings as well as any adverse events regarding human subjects.

## Discussion

This intervention is innovative because it incorporates structured simulations for both technical and communication skills training into a caregiver psychoeducational intervention. Furthermore, the delivery of the intervention parallels the trajectory of the radiation oncology patient experience – something that is rarely done in clinical research. Simulation experiences were designed for real-time (point-of-care) attention to caregiver learning needs during cancer treatment and the transition to survivorship. We hypothesize that family caregivers receiving the intervention will feel more confident with caregiving, experience less anxiety, depression, and fatigue, and report better HRQOL than those caregivers in the control group. We also hypothesize that patients whose caregivers were in the intervention group will have lower healthcare utilization costs related to hospital admissions, emergency room visits, and intravenous fluid use.

Although the study opened in December 2019, enrollment was paused from March to July 2020 due to the emergence of the COVID-19 pandemic. Several changes were made to the protocol to assure the safety of research participants, staff, and clinicians during the pandemic. A collaborative, problem-solving approach with the IRB specialist assigned to our study facilitated timely protocol modifications so that enrollment could resume as soon as restrictions were lifted in the hospital and university. Recruitment procedures were modified to allow for remote consenting of participants. This change was challenging because it involved several steps occurring during a narrow window of time as enrollment needs to occur after the consultation visit in radiation oncology but before the patient begins treatment. Eligible patients are identified in an email to the clinical staff. We engage the radiation oncology nurses in the clinic to confirm eligibility, introduce the study to the patient and caregiver, and provide them with an information packet that includes a study brochure and consent forms. After a two-day opt-out period, the research staff then follow up with the patient and caregiver via telephone to provide further information about the study and answer questions. Consent forms can be signed and mailed to the study team or submitted online. In January 2021, with safety restrictions related to the pandemic easing, we returned to completely in-person recruitment in the clinic by the research staff. To date, we have enrolled 55 patients and their family caregivers. The refusal rates are 36.0 and 23.6 % for patients and caregivers, respectively.

Safety considerations during the pandemic also necessitated more flexibility in delivery of the intervention, while maintaining scientific integrity. The number and content of the sessions were retained. The first intervention session with the caregiver, which includes hands-on simulation of technical skills, was kept as in-person with COVID-19 safety procedures. Sessions two and three, which include role play simulation, were changed to remote delivery via telephone. This change has been challenging in that one loses nonverbal communication during the session and the role play simulations are more difficult to deliver on the telephone. However, telephone delivery has reduced burden on the caregiver. If a caregiver needs additional simulation practice with technical skills, in-person visits are scheduled by the interventionist.

Lastly, the changes we made to the study protocol will need to be evaluated for their impact on validity of the study. We are tracking key information about enrollment, data collection, and intervention delivery related to the time period that they occurred. Also, the outcomes of the participants who received the fully in-person intervention pre-pandemic will need to be compared with those who received the modified intervention during the pandemic.

This study is addressing the critical need to test theoretically-based interventions to better support, educate, and train family caregivers at point-of-care. If found to be effective, use of this intervention has potential to reach all caregivers who are faced with providing complex care to their family members.

## Data Availability

Data sharing is not applicable to this article as no datasets were generated or analyzed yet.
